# *Acinetobacter baylyi* Strain BD413 Can Acquire an Antibiotic Resistance Gene by Natural Transformation on Lettuce Phylloplane and Enter the Endosphere

**DOI:** 10.3390/antibiotics11091231

**Published:** 2022-09-10

**Authors:** Valentina Riva, Giovanni Patania, Francesco Riva, Lorenzo Vergani, Elena Crotti, Francesca Mapelli

**Affiliations:** Department of Food, Environmental and Nutritional Sciences (DeFENS), University of Milan, 20133 Milan, Italy

**Keywords:** horizontal gene transfer, phyllosphere, plant microbiome, one-health, emerging organic contaminants, surfactants, water reuse

## Abstract

Antibiotic resistance spread must be considered in a holistic framework which comprises the agri-food ecosystems, where plants can be considered a bridge connecting water and soil habitats with the human microbiome. However, the study of horizontal gene transfer events within the plant microbiome is still overlooked. Here, the environmental strain *Acinetobacter baylyi* BD413 was used to study the acquisition of extracellular DNA (exDNA) carrying an antibiotic resistance gene (ARG) on lettuce phylloplane, performing experiments at conditions (i.e., plasmid quantities) mimicking those that can be found in a water reuse scenario. Moreover, we assessed how the presence of a surfactant, a co-formulant widely used in agriculture, affected exDNA entry in bacteria and plant tissues, besides the penetration and survival of bacteria into the leaf endosphere. Natural transformation frequency in planta was comparable to that occurring under optimal conditions (i.e., temperature, nutrient provision, and absence of microbial competitors), representing an entrance pathway of ARGs into an epiphytic bacterium able to penetrate the endosphere of a leafy vegetable. The presence of the surfactant determined a higher presence of culturable transformant cells in the leaf tissues but did not significantly increase exDNA entry in *A. baylyi* BD413 cells and lettuce leaves. More research on HGT (Horizontal Gene Transfer) mechanisms in planta should be performed to obtain experimental data on produce safety in terms of antibiotic resistance.

## 1. Introduction

The rise of antibiotic resistance is posing risk on a global scale for human health. Its spread has been related to the selection pressure imposed by the use of antibiotics for clinical purposes and their presence in the environment [1], where they are considered emerging organic contaminants. Contamination with sub-lethal concentrations of antibiotics can contribute to the emergence of Antibiotic Resistance Genes (ARGs) through Horizontal Gene Transfer (HGT) mechanisms into bacterial populations [2]. Accordingly, environmental bacteria displaying multi-drug resistance phenotypes against different classes of antibiotics have been found in animal, soil, and aquatic habitats [3]. Agricultural soils were indicated among the primary sources of antibiotic-resistant bacteria (ARB) threatening human health due to ARGs diffusion via manure or sewage sludge applications and the use of reclaimed wastewater for irrigation [4,5]. The plant microbiome is a nexus for the ‘One-Health’ approach, acting as a bridge connecting soil and water microbiomes to the human one through the food chain. In fact, the consumption of food products such as raw leafy vegetables is recognized among the possible main pathways for resistome diffusion [6,7]. Bacteria that usually inhabit the phyllosphere (i.e., the surfaces of the aerial parts of a plant, mainly represented by leaves) are overall considered harmless, but they might represent transient hosts with the capability to transfer ARGs to human pathogenic bacteria by HGT [8]. ARGs can accumulate on leaves from different pathways including air and soil particles [9] and are widely detected also on produces [10]. Nonetheless, evidence of HGT events within the microbiome that colonize edible plant portions generally consumed raw is still overlooked. Among HGT mechanisms, the role of natural transformation (i.e., the direct uptake and incorporation of extracellular DNA from a bacterium) is poorly studied in the context of environmental antibiotic resistance although recent evidence speaks in favor of greater importance than previously assumed [11,12]. *A. baylyi* BD413 strain, an environmental model bacterium [13] previously used for natural transformation studies [14,15], can be a useful tool to fill in this gap of knowledge. The genus *Acinetobacter* includes several human opportunistic pathogens, such as *Acinetobacter baumannii*, recognized as major causal agents of nosocomial infections and known for the propensity to develop resistance to the main groups of antibiotics [16,17]. On the other hand, different species of the *Acinetobacter* genus are typical members of the cultivable plant endophytic microbiome [7,18] and were isolated from fresh fruits and leafy vegetables, such as lettuce [19]. Recently, the opportunity to manage the microbiota composition of lettuce and other fresh produces has been proposed as a strategy to limit the invasion of potential human pathogens and to limit outbreaks related to the consumption of contaminated food [20,21]. *A. baylyi* species is able to horizontally transfer antibiotic resistance genes to *Escherichia coli* through the secretion of vesicles [17] and HGT between these two species has been recently demonstrated on lettuce leaf discs [22]. Moreover, the frequency of HGT events involving *A. baylyi* can be influenced by different environmental and chemical stresses [4,17,23].

This study was aimed at characterizing the possible acquisition of extracellular DNA (exDNA) on lettuce phylloplane by *A. baylyi* BD413 performing in planta experiments, to clarify the role of natural transformation as entrance pathway of ARGs into the epiphytic and/or endophytic bacterial community associated to leafy vegetables. Furthermore, based on the knowledge that surfactants can alter the permeability of biological membranes [24] and can increase the entrance of bacteria into leaf tissues [25], we tested the hypothesis that heptamethyltrisiloxane, a co-formulant widely used in agriculture, enhances *A. baylyi* BD413 membrane permeability, exDNA acquisition by bacteria and plant tissues, and bacterial leaf endosphere penetration.

## 2. Results

### 2.1. A. baylyi BD413 Permanence as Viable and Culturable Cells into Lettuce Leaves

**Figure 1:**
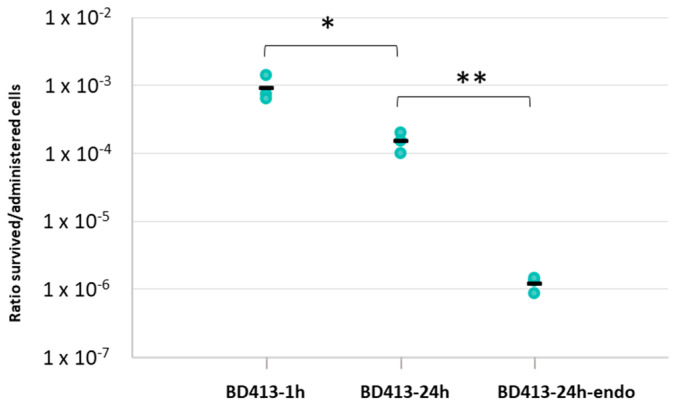
Permanence of *A. baylyi* BD413 as viable and culturable cells on lettuce phylloplane and into leaf tissues. The ratio between *A. baylyi* BD413 survived culturable cells and administered cells on lettuce phylloplane was assessed 1 h (BD413-1 h) and 24 h after spray administration (BD413-24 h). In addition, the survival into the leaf endosphere was assessed 24 h after bacterization (BD413-24 h-endo). The black line indicates the average value of three biological replicates. Stars indicate significant differences according to Student’s *t*-test (* = *p* value < 0.05; ** = *p* value < 0.001).

The capacity of the *A. baylyi* BD413 strain to survive in a viable and cultivable state on the lettuce phylloplane (i.e., the surface of leaves) was assessed after administration by spray, to simulate sprinkler irrigation. *A. baylyi* BD413 cell suspension was prepared at a concentration of 10^8^ cell/mL in physiological solution in a spray bottle. We initially evaluated the concentration of the viable and culturable bacterial cells actually released by spray (Table S1). This data was used to calculate the ratio between the CFUs/mL reisolated from the phylloplane/leaf endosphere after bacterization (i.e., survived culturable cells) and the CFUs/mL released by spray (i.e., administered cells). The experiment demonstrated that *A. baylyi* BD413 was able to adhere and survive on the leaf surface of lettuce plants (Figure 1, Figure S1) both 1 h and 24 h after bacterization (Table 1).

The table reports the results obtained for each strain 1 h and 24 h after administration on the leaf surface. Furthermore, the ratio between the *A. baylyi* BD413 cells that penetrate and survived into the endosphere and those initially administered on the leaf surface has been calculated.

However, after 24 h, the ratio between survived and administered *A. baylyi* BD413 cells showed a significantly lower value (Student’s *t*-test, *p* = 0.0335) than the one obtained 1 h after administration. *A. baylyi* BD413 cells were reisolated also from the leaf endosphere 24 h after bacterization (Table 1). The ratio between the survived cells reisolated from the leaf tissues and those initially administered on the leaf surface was significantly lower (Figure 1) compared to that measured on the phylloplane at the same time point (Student’s *t*-test, *p* = 0.0066).

The ability of *A. baylyi* BD413 to survive on the lettuce phylloplane was compared with those of the laboratory strain *E. coli* DH5α and the lettuce strain *K. cowanii* VR04 (Figure S2) as a benchmark to clarify its possible adaptation to this ecological niche. The CFUs/mL released by spray on lettuce leaves were measured for *E. coli* DH5α and *K. cowanii* VR04 (Table S1) to determine also for these strains the ratio between the survived and the administered cells on the leaf surface. According to ANOVA tests (Table S2), at both the experimental times the survival of *A. baylyi* BD413 into a viable and culturable state was not significantly different in comparison to *E. coli* DH5α values (Table 1). The survival of *K. cowanii* VR04 on phylloplane (Table 1) was significantly higher compared to *A. baylyi* BD413 1 h after the strain administration, while it decreased after 24 h to a value (Table 1) not significantly different from that recorded for *A. baylyi* BD413 (Figure S1, Table S2). For all survival experiments, bacterial colonies were reisolated and identified by ITS fingerprinting as *A. baylyi* BD413 (Figure S1a–c), *E. coli* DH5α (Figure S1d), and *K. cowanii* VR04 (Figure S1e).

### 2.2. Natural Transformation on Nitrocellulose Membrane Filters

**Figure 2:**
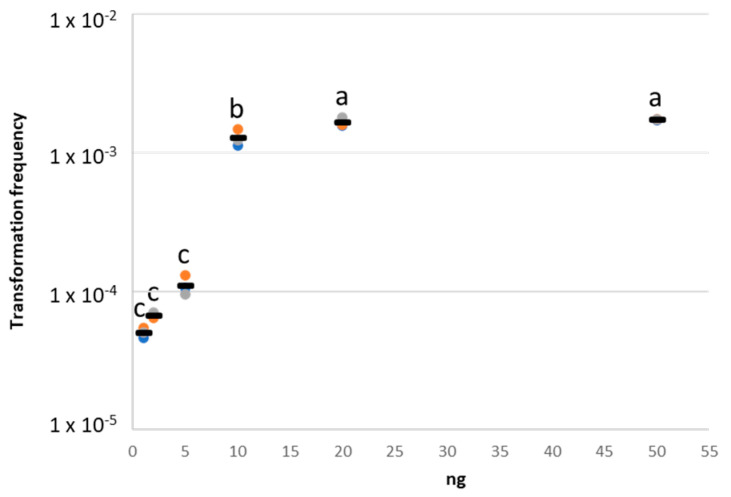
Selection of extracellular DNA quantities for in vivo *A. baylyi* BD413 natural transformation assays. Transformation frequency of *A. baylyi* BD413 was calculated by performing an in vitro natural transformation experiment on nitrocellulose membrane filters using a range of quantities of plasmid pZR80(gfp), chosen according to the literature information about exDNA concentration in wastewaters. The black line indicates the average value of three technical replicates. Different letters (a, b, c) indicate significant differences between the values of transformation frequency measured using DNA quantities ranging from 1 to 50 ng, according to the Tukey-Kramer post-hoc test (ANOVA, *p*-value < 0.0001).

To set up the best condition for the in vivo natural transformation assay, plasmid DNA acquisition by *A. baylyi* BD413 was initially tested on nitrocellulose membrane filters using different quantities of pZR80(gfp), comprised between 1 and 50 ng based on literature data available about exDNA concentration in wastewaters [26]. Using 1, 2, and 5 ng of plasmid DNA per transformation assay, we measured not significantly different transformation frequency values (Figure 2, Table S3). A significantly higher (ANOVA, Tukey-Kramer post-hoc test; *p* < 0.0001) transformation frequency was obtained using 10 ng of pZR80(gfp) (Figure 2, Table S3). This value further and significantly increased (Figure 2, Table S3) when performing the experiment with 20 ng and 50 ng of plasmid.

**Figure 3:**
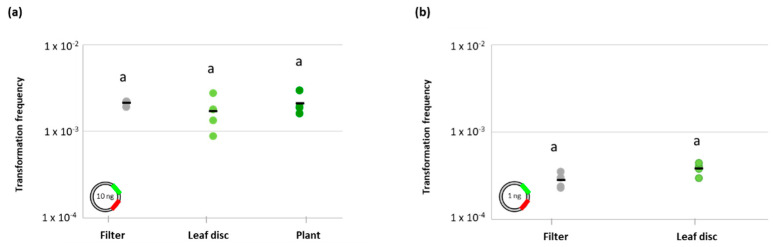
Natural transformation of A. baylyi BD413 under different experimental setups. Transformation frequency of *A. baylyi* BD413 was calculated using 10 ng (**a**) and 1 ng (**b**) of plasmid pZR80(gfp) on nitrocellulose membrane filters, leaf discs, and in planta. For all conditions, the black line indicates the average value of four biological replicates. The same letter (a) indicates no significant differences according to (**a**) ANOVA test (*p*-value > 0.05) or (**b**) Student’s *t*-test (*p*-value > 0.05).

Based on these results we selected the lowest quantities of plasmid DNA suitable for subsequent in vivo tests, namely 1 ng and 10 ng, which represents a compromise between the environmental conditions and the detection of natural transformation events [26]. Once the plasmid quantities have been selected, the *A. baylyi* BD413 natural transformation experiment on nitrocellulose membrane filters was repeated using four biological replicates (i.e., four different cultures of *A. baylyi* BD413) to obtain results statistically comparable with those subsequently generated on lettuce leaves. In the latter assay, the *A. baylyi* BD413 transformation frequency showed a value of 2.12 × 10^−3^ ± 1.43 × 10^−4^ using 10 ng of plasmid (Figure 3b), while this value decreased to 2.80 × 10^−4^ ± 5.65 × 10^−5^ performing the in vitro experiment with 1 ng of DNA (Figure 3b).

### 2.3. Natural Transformation on Lettuce Leaves

The capability of *A. baylyi* BD413 to acquire exDNA on lettuce leaf surface has been tested using both leaf discs and in planta. Using 10 ng of pZR80(gfp) plasmid, a transformation frequency of 1.70 × 10^−3^ ± 7.98 × 10^−4^ was measured as the average value of four biological replicates (i.e., four leaf discs) (Figure 3a). Using the same amount of plasmid, the analysis was then conducted in planta on four biological replicates (i.e., four leaves of the same lettuce plant) and an average transformation frequency of 2.09 × 10^−3^ ± 5.94 × 10^−4^ was observed (Figure 3a). The same test was repeated during a second independent experiment using a different lettuce plant, measuring not statistically different values of average transformation frequencies (9.66 × 10^−3^ ± 1.59 × 10^−2^, Student’s *t*-test, *p* = 0.3774). Using this quantity of plasmid, the values of *A. baylyi* BD413 transformation frequencies detected on lettuce leaf discs and lettuce plant were not significantly different compared to that observed in vitro (ANOVA, *p* = 0.5411; Figure 3a).

The natural transformation assays on the leaf surface were repeated using a lower quantity of exDNA, namely 1 ng of the pZR80(gfp) plasmid, and comparing the results with those obtained in vitro. According to statistical analysis, the transformation frequencies of *A. baylyi* BD413 obtained on lettuce leaf discs (3.81 × 10^−4^ ± 6.20 × 10^−5^) and on nitrocellulose membrane filters (2.80 × 10^−4^ ± 5.65 × 10^−5^) did not show significant differences (Student’s *t*-test, *p* = 0.0527) even using 1 ng of exDNA (Figure 3b). When the experiment was conducted in planta with the same quantity of plasmid, the transformation events approximate the detection limit. While total CFUs were still countable (on the serial dilution 10^−3^), CFUs derived from transformant cells were not detectable using 1 ng of exDNA, even plating the undiluted samples, for three out of the four biological replicates. Transformant cells are a fraction of the total *A. baylyi* BD413 culturable cells, hence this data agrees with the overall decrease of two orders of magnitude presented by *A. baylyi* BD413 total CFUs values in planta compared to those obtained from the leaf disc test. Moreover, we cannot exclude that this result was directly due to the lower DNA quantity used, which could undergo a quicker degradation in planta under greenhouse conditions. We could detect transformants *A. baylyi* BD413 colonies only for one of the biological replicates (transformation frequency = 2.94 × 10^−5^ ± 1.47 × 10^−5^, expressed as the average value of three technical replicates), a result that hampers the statistical comparison of in planta results with those observed in vitro and on leaf discs (Figure 3b).

Negative control samples (i.e., leaf discs inoculated with *A. baylyi* BD413 without plasmid) were included in all natural transformation assays, and no transformant colonies were detected on LB agar supplemented with 100 µg/mL kanamycin, confirming the reliability of the presented results. Moreover, ITS-PCR confirmed the identity of the reisolated colonies (Figure S3a,c,e), and gfp-PCR confirmed the plasmid acquisition by *A. baylyi* BD413 transformants (Figure S3b,d,f), while the gfp expression was assessed by epifluorescence microscopy (Figure S3g). The detected natural transformation events occurred on the leaf surface, and not in liquid before leaf inoculation, as demonstrated by the absence of kanamycin-resistant colonies at the end of a control experiment performed incubating in physiological solution an *A. baylyi* BD413 bacterial suspension with plasmid pZR80(gfp), at room temperature for 30 min.

### 2.4. Effect of Surfactant Molecule on the Ability of A. baylyi BD413 to Acquire exDNA and Enter the Leaf Endosphere

**Figure 4:**
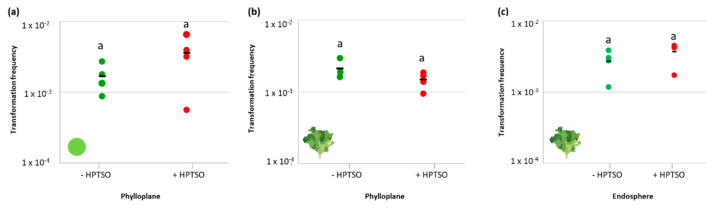
Influence of the heptamethyltrisiloxane surfactant on the natural transformation of *A. baylyi* BD413. Transformation frequency of *A. baylyi* BD413 with 10 ng of plasmid pZR80(gfp) on leaf disc (**a**) and in planta (**b**) in the presence (+HPTSO) or absence (-HPTSO) of the surfactant molecule heptamethyltrisiloxane. Panel (**c**) indicates the ratio between transformant and total *A. baylyi* BD413 colonies reisolated from the leaf endosphere. The black line indicates the average value of four biological replicates. The same letter (a) indicates no significant differences between the values detected in the presence or absence of HPTSO (Student’s *t*-test, *p*-value > 0.05).

Once verified that heptamethyltrisiloxane (HPTSO, 0.021% *v*/*v*) does not cause inhibitory effects on *A. baylyi* BD413 growth, its possible influence on bacterial transformation frequency on leaf surface was tested on leaf disc and in planta using 10 ng of plasmid pZR80(gfp). When HPTSO was administered on leaf surface together with *A. baylyi* BD413, the transformation frequencies on leaf disc and in planta were 3.6 × 10^−3^ ± 2.5 × 10^−3^ and 1.47 × 10^−3^ ± 3.95 × 10^−4^, respectively (Figure 4a,b). Thus, in both the experimental setups, the ability of *A. baylyi* BD413 to acquire exDNA did not result significantly different in the presence and absence of HPTSO (Student’s *t*-test, *p*-value > 0.05).

The experiment was repeated in planta to reisolate total and transformant colonies of *A. baylyi* BD413 from the leaf endosphere and measure their ratio in the presence and absence of HPTSO (Figure 4c), obtaining values that were not significantly different (3.71 × 10^−3^ ± 1.33 × 10^−3^ and 2.71 × 10^−3^ ± 1.11 × 10^−3^, respectively, Student’s *t*-test, *p* = 0.2919). Identity and plasmid acquisition by the putative *A. baylyi* BD413 transformants isolated from leaf tissues were confirmed by ITS-PCR and gfp-PCR (Figure S4). The effect of the tested surfactant on the permeability of *A. baylyi* BD413 cell membrane was assessed by measuring the changes in both the internal and the total cell membrane permeability in presence of HPTSO. The bacterial cell permeability was not influenced by the presence of the surfactant in the growth medium at the considered time points (Figure S5), coherently with the lack of increased natural competence (Figure 4a,b).

**Figure 5:**
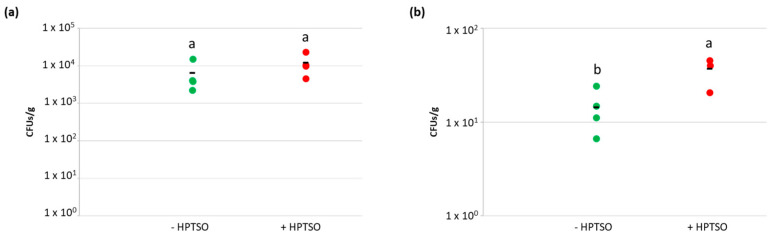
Entry and survival of culturable *A. baylyi* BD413 cells in lettuce endosphere. Number of total (**a**) and transformant (**b**) *A. baylyi* BD413 CFUs/g of leaf reisolated from lettuce endosphere in presence (+HPTSO) or absence (-HPTSO) of the surfactant molecule heptamethyltrisiloxane. The black line indicates the average value of four biological replicates. In each panel, different letters (a, b) indicate significant differences of the CFUs/g of *A. baylyi* BD413 detected in the presence/absence of HPTSO, according to Student’s *t*-test (*p*-value > 0.05).

Considering that the application of surfactant molecules may enhance the internalization of bacteria into lettuce leaves, by performing the natural transformation experiment in planta we also aimed at measuring the concentration of *A. baylyi* BD413 total and transformant colonies in the lettuce leaf tissues. As hypothesized, the concentrations of total and transformant *A. baylyi* BD413 colonies in the lettuce endosphere showed higher values in leaves treated with HPTSO (Figure 5) and such difference was statistically significant for transformant colonies (Student’s *t*-test, *p* = 0.0149). The latter result could be related to a higher uptake of exDNA by plant tissues in the presence of HPTSO resulting in the occurrence of transformation events directly in the endosphere. To test such a hypothesis, the concentration of pZR80(gfp) plasmid in the lettuce leaves was measured both in the presence and absence of the surfactant, providing the exDNA at the same quantity used for the natural transformation in planta (i.e., 10 ng which corresponded to 1.5 × 10^9^ copies of pZR80(gfp) plasmid). The qPCR results showed that the gfp copy number per gram of leaf was higher when the exDNA was provided on the leaves together with HPTSO (1.60 × 10^7^ ± 2.25 × 10^7^) although the comparison with data measured in the absence of HPTSO (3.56 × 10^6^ ± 9.47 × 10^5^) revealed that the difference was not statistically significant (Student’s *t*-test, *p* = 0.3138; Figure S6).

## 3. Discussion

A recent work [22] demonstrated that *A. baylyi* can transfer plasmid DNA carrying ARGs to *E. coli* clinical isolates on leaf discs and that the *E. coli* transformant cells can subsequently colonize the mouse gut and transfer the antibiotic resistance determinants to *Klebsiella pneumoniae* in vivo. In this context, the present study investigated one of the possible upstream steps, namely the acquisition of exDNA by *A. baylyi* BD413 in planta by natural transformation, using lettuce as a model leafy vegetable. Literature data indicate in fact that *Acinetobacter* spp. are abundant members of water, soil, phyllosphere, and endosphere microbiome [18,27,28], and the species *A. baylyi*, among others affiliated to this genus, was isolated from commercial lettuce [19]. During this study, we verified the ability of *A. baylyi* strain BD413 to survive on the lettuce leaf surface and enter the internal tissues when administered in a physiological solution simulating spray irrigation. The administration method was chosen considering that sprinklers are irrigation systems widely used for both field and greenhouse leafy vegetable farming. The capacity of *A. baylyi* BD413 to remain viable and culturable after spray administration was considered a prerequisite to allowing its use as a model bacterium for HGT experiments in planta. The results of the permanence assay showed that *A. baylyi* BD413 can survive on the phylloplane 1 day after administration at concentrations that were comparable to that of the lettuce strain *K. cowanii* VR04, confirming the adaptation of the genus *Acinetobacter* to the phyllosphere ecosystem [28]. This is in agreement with the previously reported capacity of *A. baylyi* to grow as an epiphytic bacterium on lettuce leaves, due to the possible use of leaf exudates as a carbon source [15].

In this study, for in vivo natural transformation, we selected two exDNA quantities (i.e., 1 and 10 ng) that mimic the environmental concentration detected in wastewater [26]. Indeed, water reuse for irrigation purposes is considered a priority, in relation to the occurrence of water shortage periods that affect crop productivity on a global scale [29]. In this framework, the recent EU legislation on water reuse aims at regulating the concentration of several emerging contaminants, including antibiotic resistance determinants in treated wastewater used for irrigation. However, the data available on antibiotic resistance in wastewater treatment plants, their effluents, and the agri-food systems, generally refer to the abundance and/or distribution of ARGs and ARB [30]. On the contrary, the mechanisms of ARG diffusion and the anthropogenic input that might increase their frequency are rarely analyzed.

Here, we demonstrated that the natural transformation of *A. baylyi* BD413 occurs on the lettuce phylloplane at the same frequency that is encountered in the laboratory applying optimal conditions in terms of temperature, nutrient provision, and absence of microbial competitors. Notably, this result was obtained not only on leaf disc but also during in planta experiment. These data corroborate previous evidence that nutrient limitation does not act in *A. baylyi* as a factor enhancing natural competence, differently from what is reported for other bacteria such as *Haemophilus influenzae* [27]. To the best of our knowledge, no previous data are available on the natural transformation frequency of environmental bacteria in planta on lettuce phylloplane. Hence, this study can add an important piece of knowledge for the risk assessment of ARGs diffusion by HGT and their entrance into the food supply chain. The frequency of HGT events in the environment could be influenced by several factors. For instance, bacterial exposure to water disinfection products used in wastewater treatment plants, such as bromoacetic acid and sodium hypochlorite, was demonstrated to enhance the process of transformation in naturally competent bacteria, promoting ARGs spread [23,31]. In this study, the possible influence of a surfactant molecule (i.e., heptamethyltrisiloxane) on *A. baylyi* BD413 natural transformation frequency was investigated. Since surfactants allow a more uniform spread of the agrochemicals over plant surfaces, they are widely used in agriculture as co-formulants in the commercial preparation of many pesticides and fertilizers provided through foliar application [25]. This class of molecule has been chosen as representative of environmental conditions that could be easily found in the field. In addition, biosurfactants can be produced by epiphytic bacteria as a strategy to move on the leaf surface towards areas where nutrients are more abundant [32]. Though the results of this study showed that heptamethyltrisiloxane did not significantly change the frequency of *A. baylyi* BD413 natural transformation, higher bacterial concentrations were observed in both reisolating wild-type and transformant *A. baylyi* BD413 cells from the lettuce endosphere, the latter retrieved at concentrations significantly higher in presence of the surfactant. The hypothesis that the higher concentration of transformant *A. baylyi* BD413 cells could be related to a higher uptake of exDNA by the leaf tissues allowing natural transformation directly in the leaf endosphere was not confirmed by the data. However, this process could be not excluded since a higher, still not significant, concentration of the pZR80(gfp) plasmid was measured in the presence of HPTSO. Regardless of the presence of the surfactant, the results of this study showed in fact that a high amount of the provided exDNA was detected in the leaf. Future studies could be focused on exDNA fate and potential to be acquired by the endophytic bacterial populations, considering the lack of overall data in the literature, where the role of exDNA is considered solely in the frame of plant-microbe interaction and plant immune response stimulation [33].

The use of Silwet L-77, a commercial product whose main component is the surfactant molecule tested in this study, was previously demonstrated to increase the entrance of a human pathogen, i.e., *Salmonella enterica*, in tomato leaf tissues and fruits [25]. Moreover, the foliar application of other commercial products, such as vegetable-derived bioactive compounds, can alter the microbiome composition of lettuce leaves promoting the growth of certain bacterial genera, including *Acinetobacter* [34]. The fact that *A. baylyi* BD413 can penetrate the lettuce leaf endosphere, where its viable and culturable populations were retrieved, is an important aspect in terms of food safety considering that once bacterial cells enter the leaf tissues they cannot be removed by washing and disinfection procedures. Since HGT between *A. baylyi* and members of the family *Enterobacteriaceae* was demonstrated [22], the relevance of the endophytic lifestyle of *A. baylyi* BD413 raises considering the frequent occurrence of *Enterobacteriaceae* in the edible tissues of lettuce [35], where they are three times more abundant compared to the root system [36].

## 4. Materials and Methods

### 4.1. A. baylyi BD413 Survival Assay on Lettuce Phylloplane and into Leaf Endosphere

*Acinetobacter baylyi* BD413, a strain resistant to rifampicin, was used for the bacterization of *Lactuca sativa* (var. Canasta) plants to verify its ability to survive in a viable and culturable state on lettuce phylloplane (i.e., leaf surface) and enter the leaf endosphere. *A. baylyi* BD413 was grown in Luria-Bertani (LB) medium (Sigma-Aldrich, St. Louis, MO, USA) supplemented with rifampicin (100 µg/mL, Sigma-Aldrich) for 24 h at 30 °C in an orbital shaker. The total cell number of the bacterial suspensions was evaluated at the optical microscope (Motic, BA310E) using a Thoma chamber. *A. baylyi* BD413 bacterial culture was centrifuged twice at 4000 rpm for 10 min and the cells were re-suspended in sterilized physiological solution (i.e., NaCl 0.9%) to obtain a final bacterial concentration of 10^8^ cell/mL. The bacterial suspension was transferred in a spray bottle and dispensed on lettuce leaves. *A. baylyi* BD413 was sprayed (1 spray per leaf, corresponding to a volume of 100 µL) on five leaves per plant (number of bacterized plants = 6). Six plants were treated with a sterilized physiological solution and used as a negative control. To define the number of bacterial CFUs released on each plant from the spray bottle, five sprays were collected in a sterile tube, serially diluted, and plated in triplicates on LB agar medium supplemented with 100 µg/mL rifampicin.

Bacterized and control plants were kept in greenhouse at 25 °C. After 1 h, the epiphytic bacteria were recovered from negative control plants (n = 3) and bacterized plants (n = 3): the five treated leaves per plant were removed with a sterile scalpel and put in 20 mL of sterilized physiological solution for 1 h under shaking to detach bacterial cells from the leaf surface. Cell suspensions were serially diluted, plated in triplicate on LB agar medium supplemented with rifampicin (100 µg/mL) and CFUs were counted after incubation at 30 °C for 24 h. In addition, epiphytic *A. baylyi* BD413 cells were recovered, as described above, 24 h after plant bacterization from the remaining bacterized (n = 3) and negative control (n = 3) plants. From the latter plants, once the epiphytic *A. baylyi* BD413 cells were detached for isolation, lettuce leaves were surface sterilized with ethanol 70% for 30 sec and rinsed three times with sterilized distilled water for 3 min, to isolate *A. baylyi* BD413 cells from the leaf endosphere (protocol adapted from reference [37]). A 100 µL sample of water from the last rinsing step was plated on LB agar medium supplemented with 100 µg/mL rifampicin to confirm the complete removal of *A. baylyi* BD413 from the phylloplane. Finally, leaves were smashed using sterile mortar and pestle in physiological solution, serially diluted, and plated in triplicate on LB agar medium supplemented with 100 µg/mL rifampicin. The survival ability of *A. baylyi* BD413 was measured as the fraction between the CFUs/mL re-isolated from the phylloplane/leaf endosphere after bacterization and the CFUs/mL released by spray.

To confirm the identity of the isolates, bacterial colonies isolated from the phylloplane (n = 10) and from the leaf endosphere (n = 10) of each bacterized lettuce plant were streaked and the DNA was extracted from each colony through boiling cell lysis. The 16–23 S rRNA Intergenic Transcribed Spacer (ITS) region was amplified and isolate identification was assessed by ITS-PCR fingerprinting [38] comparing the ITS profiles of the isolated bacteria with that of the *A. baylyi* BD413 strain inoculated on lettuce.

The ability of the *A. baylyi* BD413 strain to survive on the lettuce phylloplane was compared with those of other bacteria. The described experimental procedure was adopted for the rifampicin-resistant mutants of the laboratory *Escherichia coli* strain DH5α and of the *Kosakonia cowanii* VR04, a bacterial strain previously isolated from lettuce leaf endosphere using the Violet Red Bile Lactose Agar medium (Sigma-Aldrich; Mapelli F., personal communication). Rifampicin-resistant mutants were prepared according to a published protocol [39].

### 4.2. Natural Transformation Protocols

#### 4.2.1. Bacterial Culture Preparation

*A. baylyi* BD413 strain was grown in 20 mL of LB liquid medium overnight at 30 °C under shaking, subsequently inoculated in a ratio of 1:100 *v/v* in LB medium, and incubated at 30 °C until the cells reached the early exponential growth phase, corresponding to optical density (OD) value of 0.4–0.5 at 600 nm (UV/VIS Spectrophotometer 7305, Jenway, London, UK). The bacterial cells were then centrifuged at 4000 rpm for 10 min and re-suspended in sterilized physiological solution to obtain a final bacterial concentration of 10^9^ cell/mL. Four aliquots of 100 µL of cells were prepared and the proper quantity of extracellular DNA (exDNA) was added and gently mixed. For each experiment, the fifth aliquot of cells was used as negative control (no DNA was added).

#### 4.2.2. Extracellular DNA (exDNA) Preparation

Natural transformation experiments were conducted using the pZR80(gfp) plasmid as extracellular DNA. The plasmid harbors a kanamycin resistance gene (aphA-3) and a gene codifying for the green fluorescent protein (gfp) as an optical marker [14]. The plasmid was previously extracted from an overnight culture of the strain *E. coli* (pZR80(gfp)) using the QIAPrep Spin Miniprep Kit (Qiagen, Hilden, Germany) following the manufacturer’s instructions and its concentration was assessed fluorometrically using the Qubit™ dsDNA HS Assay Kit (Thermo Fisher Scientific, Waltham, MA, USA).

#### 4.2.3. Selection of the exDNA Quantity for In Vivo Experiments

To determine the quantity of exDNA to be used for in vivo natural transformation experiments, a preliminary in vitro test was conducted in triplicate using 1, 2, 5, 10, 20, and 50 ng of the pZR80(gfp) plasmid and nitrocellulose membrane filters (GSWP, 25 mm diameter, 0.22 mm pore size, Millipore, Burlington, MA, USA). The bacterial suspension (10^9^ cell/mL) was mixed with the different plasmid quantities (in a final volume of 500 µL) and placed on a sterile nitrocellulose membrane filter, previously positioned on LB agar plates. After 24 h incubation at 30 °C, the cells were detached from the filter by resuspension in 1 mL of physiological solution, serially diluted, and plated on both LB agar and LB agar supplemented with 100 µg/mL kanamycin (Sigma-Aldrich) to count the total and the transformant CFU numbers present on the filter, respectively. Transformation frequency was calculated as the number of kanamycin-resistant transformant colonies over the total number of colonies. Randomly selected transformant colonies (n = 10) were checked by epifluorescence microscopy (Zeiss Axio Lab.A1) for the expression of the gfp gene harbored on the pZR80(gfp) plasmid.

#### 4.2.4. Natural Transformation on Leaf Disc

The lowest DNA quantity that allows the detection of the transformation events on nitrocellulose membrane filters was chosen for in vivo experiments. The ability of *A. baylyi* BD413 to acquire exDNA was tested on the surface of lettuce (*Lactuca sativa* var. Canasta) leaf discs, collected using a round-shape cutting of 4.5 cm diameter, sterilized by dipping in ethanol 70% and placed in empty 60 mm diameter Petri dishes. A solution of 10^9^ cell/mL bacterial cells and plasmid (or without plasmid DNA in the case of negative control) was prepared in a final volume of 100 µL and placed on the leaf disc surface and, after 24 h of incubation at 30 °C, leaf discs were placed in sterile tubes with 1 mL of physiological solution to detach the cells. The bacterial cell suspension was serially diluted and plated on LB agar and LB agar supplemented with 100 µg/mL kanamycin. After 24 h at 30 °C, CFUs were counted and the transformation frequency was calculated. Randomly selected transformant colonies (n = 30) were checked for (i) their identity through ITS-PCR fingerprinting using *A. baylyi* BD413 as positive control and (ii) the presence of the gfp gene harbored by the pZR80(gfp) plasmid. The gfp gene was amplified by PCR using the primers GFP540F (5′-CAAGAGTGCCATGCCCGAAGG-3′) and GFP875R (5′-GGTAAAAGGACAGGGCCATCGCC-3′) [40] and the following thermal protocol: 95°C for 4 min, followed by 35 cycles of 95 °C for 45 s, 60 °C for 1 min and 72 °C for 1 min and a final extension at 72 °C for 10 min.

#### 4.2.5. Natural Transformation in Planta

Natural transformation of *A. baylyi* BD413 in presence of pZR80(gfp) plasmid was tested in planta using lettuce plants (*Lactuca sativa* var. Canasta) under greenhouse conditions. The experiment was conducted using four replicates, corresponding to four leaves of the same lettuce plant: the four leaves were inoculated with 10^9^ cell/mL *A. baylyi* BD413 cell suspension mixed with 10 ng of the pZR80(gfp) plasmid (final volume of 100 µL). In addition, one leaf was inoculated with cell suspension (no plasmid, negative control) to assess the absence of native kanamycin-resistant bacteria on the lettuce phylloplane. Each bacterized leaf was covered using a sterile empty Petri dish to avoid environmental contamination from the greenhouse. After 24 h, the inoculated leaves were removed from the plant with a sterile scalpel and kept in the Petri dishes, where 1 mL of physiological solution was added to detach the cells from the leaf surface. After shaking, bacterial cell suspensions were serially diluted and plated on LB agar and LB agar supplemented with 100 µg/mL kanamycin to reisolate total and transformant *A. baylyi* BD413 colonies. CFUs were counted after incubation at 30 °C for 24 h and the transformation frequency was calculated. This experiment was repeated by applying the same conditions to reisolate total and transformant *A. baylyi* BD413 colonies from the leaf endosphere, after leaf surface sterilization. At the end of both experiments, the identity and the presence of the gfp gene were checked on randomly selected transformant colonies (n = 30) as described in the previous paragraph. To demonstrate that the HGT events occurred in planta and not in liquid (i.e., before the suspension of bacterial cells and plasmid were inoculated on the leaves), we incubated the bacterial suspension with pZR80(gfp) plasmid at room temperature for 30 min and plated the cells on LB agar medium supplemented with 100 µg/mL kanamycin. After incubation at 30 °C for 24 h, the presence of transformant colonies was checked on the Petri dishes.

### 4.3. Entry of Total and Transformant A. baylyi BD413 Cells into Leaf Endosphere

The ability of total and transformant cells of *A. baylyi* BD413 to enter the endosphere of lettuce leaves was measured during the in planta experiment described in the previous paragraph. After the removal of cells from the leaf surface, lettuce leaves were surface sterilized and smashed as described in the survival assay (see Section 4.1). Serial dilutions were prepared and plated on LB agar and LB agar supplemented with 100 µg/mL kanamycin. A 100 µL sample of water from the last rinsing step was plated on LB agar medium with and without 100 µg/mL kanamycin to confirm the complete removal of *A. baylyi* BD413 transformant and total cells from the phylloplane. After 24 h at 30 °C, the number of total and transformant CFUs present in the leaf endosphere were counted and expressed as CFUs/g of leaf tissue. All putative transformant colonies were checked for their identity and the presence of the gfp gene as described in Section 4.2.4.

### 4.4. Effect of a Surfactant Molecule on A. baylyi BD413 Transformation and Penetration into the Endosphere

Heptamethyltrisiloxane (HPTSO, Merck, Kenilworth, NJ, USA) was selected as a representative surfactant molecule for the experiment. HPTSO is the principal component (85%) of Silwet L-77, an organo-silicone surfactant used in agriculture for foliar applications of many agro-chemical products, including herbicides, insecticides, fungicides, plant growth regulators, and fertilizers with a concentration comprised between 0.025–0.1% [25]. In this experiment HPTSO was diluted in sterilized physiological water and used at 0.021% *v*/*v*, corresponding to its final concentration for foliar application on leafy green vegetables in the field, according to the Silwet L-77 product label. Firstly, the possible inhibitory effect of the surfactant on *A. baylyi* BD413 growth was analyzed in triplicate both in solid and liquid LB medium supplemented by 0.021% *v*/*v* HPTSO. Then, the effect of HPTSO on the transformation frequency of *A. baylyi* BD413 was tested on leaf discs and in planta mixing the surfactant molecule with *A. baylyi* BD413 and plasmid DNA prior to spotting the bacterial suspension on lettuce leaves (n = 4), and following the procedure described in the previous paragraph to reisolate total and transformant *A. baylyi* BD413 cells from the leaf surface. Moreover, the HPTSO influence on the *A. baylyi* BD413 ability to penetrate the leaf tissues was tested by reisolating total and transformant colonies from the endosphere, using LB agar and LB agar supplemented with 100 µg/mL kanamycin, respectively, and calculating their CFU number per gram of leaf tissue.

### 4.5. Bacterial Cell Membrane Permeability Assays

The effect of HPTSO on *A. baylyi* BD413 membrane permeability was determined through two different methods. The first one measures the permeability of the inner membrane and it is based on an aqueous hydrolysis reaction of o-nitrophenyl-β-Dgalactopyranoside (ONPG, Merck) [41]. The second method allows for the determination of the total cell membrane permeability using a crystal violet solution as previously described [42].

In detail, the inner membrane permeability assay was conducted growing *A. baylyi* BD413 in LB liquid medium added with 2% lactose at 30 °C overnight. Cells were recovered by centrifugation (4500× *g* for 10 min) and resuspended in physiological solution to a final concentration of 10^8^ cell/mL, ONPG 2.5 mM, and HPTSO (0.021% *v*/*v*). Three biological replicates were prepared and the other three replicates without the addition of HPTSO were used as control. The samples were incubated at 30 °C and after 2, 7, and 24 h the sample OD at 415 nm was measured (UV/VIS Spectrophotometer 7305, Jenway) to monitor the production of o-nitrophenol over time.

For the determination of the total membrane permeability, *A. baylyi* BD413 was grown in LB liquid medium overnight and resuspended to a final concentration of 10^8^ cell/mL in physiological solution after centrifuging the cell culture at 4500× *g* for 10 min. HPTSO (0.021% *v*/*v*) was added to three biological replicates while three samples without the surfactant molecule were used as control. Samples were incubated at 30 °C and, after 6- and 24-h, cells were harvested at 9300× *g* for 5 min and resuspended in a physiological solution containing 5 µg/mL crystal violet. Cell suspensions were incubated at 30 °C for 10 min and centrifuged at 13,400× *g* per 15 min. The OD of the supernatants was measured at 590 nm (UV/VIS Spectrophotometer 7305, Jenway). The OD value of the crystal violet initial solution used in the assay was considered 100%. The percentage of crystal violet uptake was calculated using the following formula: (OD value of the sample)/(OD value of the crystal violet solution) × 100 [42].

### 4.6. Lettuce Leaf Acquisition of Extracellular DNA

Acquisition of pZR80(gfp) plasmid by lettuce plants (*Lactuca sativa* var. Canasta) was tested. The experiment was conducted using four replicates (i.e., four leaves) per treatment, and each treatment was conducted on a separate plant. Leaf treatments were as follows: (i) addition of 10 ng of the pZR80(gfp) plasmid (final volume of 100 µL), (ii) addition of 10 ng of the pZR80(gfp) plasmid and HPTSO (0.021% *w*/*v*). Lastly, four replicates were put in contact with 100 µL of sterile water to serve as a negative control.

After 24 h, the treated leaves were removed from the plant with a sterile scalpel and kept in Petri dishes. Before DNA extraction from leaves, the fresh weight was measured, and surface sterilization was performed as reported in Section 4.1. Each leaf was separately crushed in a sterile mortar by N_2_ liquid addition, the biological material was then collected using a sterile spatula and stored at −20 °C.

Subsequent DNA extraction from the 16 leaves was conducted by using DNeasy Plant Mini Kit (Qiagen), according to manufacturer instructions, adding an initial step of plants’ material destruction by the TissueLyser II (Qiagen) to obtain higher yields of DNA. Qubit dsDNA HS kit (Invitrogen, Waltham, MA, USA) was used for the DNA concentration measurement of each sample. The DNA extracted from the leaf tissues was used as a template to perform a PCR to assess the presence of the pZR80(gfp) plasmid. The PCR targeted a fragment (1100 bp) of pZR80(gfp) plasmid including both the gfp and the aph-A genes, which was amplified using the primers GFP540F (5′-CAAGAGTGCCATGCCCGAAGG-3′) and aphA-3R2 (5′- ACTCTTCCGAGCAAAGGACG-3′), and the following thermal protocol: 95 °C for 4 min, followed by 35 cycles of 95 °C for 45 s, 59 °C for 1 min and 72 °C for 2 min and a final extension at 72 °C for 10 min. Quantitative PCR (qPCR) reactions of the gfp gene were conducted according to a published protocol [40], with slight modifications. The reactions were executed in polypropylene 96-well plates on a BIORAD CFX Connect™ Real-Time PCR Detection System by the amplification of the gfp sequence, using primers 540F (5′-CAAGAGTGCCATGCCCGAAGG-3′) and 875R (5′-GGTAAAAGGACAGGGCCATCGCC-3′) with the following conditions: 0.2 µM of each primer, 2x SsoAdvanced™Universal SYBR^®^Green Supermix (BIORAD), 1 µL DNA template, 12 µL final volume. The reaction conditions consisted of three-step cycles of 45 s at 98 °C and 30 s at 60 °C and 30 s at 72 °C, for a total of 40 cycles. Each plate included triplicate reactions per DNA sample and the appropriate set of standards. Melting curve analysis was conducted following each assay to confirm the amplification of specific PCR products and the number of gfp copies was related to the leaves’ weights of each biological sample.

### 4.7. Statistical Analyses

The results of the *A. baylyi* BD413 survival assay on leaf surface at different time points and comparing epiphytic vs. endophytic permanence (24 h after administration) were analyzed statistically by Student’s *t*-test.

The ratio between survived culturable cells and administered cells shown by *A. baylyi* BD413, *E. coli* DH5α, and *K. cowanii* VR04 were compared by ANOVA applying a post-hoc Dunnett’s test considering *A. baylyi* BD413 as the control thesis. Natural transformation frequencies detected on nitrocellulose filters using different DNA quantities were analyzed by ANOVA applying a Tukey-Kramer post-hoc test. Likewise, the *A. baylyi* BD413 transformation frequencies detected on nitrocellulose filters, lettuce leaf discs, and in planta using 10 ng of plasmid pZR80(gfp) were analyzed by ANOVA. ANOVA and post-hoc tests indicated above were performed using JMP Pro 16 Software. All the other results related to natural transformation frequencies (e.g., in the presence and absence of HPTSO) were analyzed by a Student’s *t*-test using the Microsoft Excel software. To evaluate the influence of surfactant treatment on DNA acquisition by plant tissues, the differences of gfp copy number/gram of leaf tissue were firstly checked with a Dunnett’s test against the control non-treated plants using the package DescTools with the R software version 4.2.0 and then a Student’s *t*-test was performed between the two treatments.

## 5. Conclusions

This study highlights that natural transformation is an HGT mechanism occurring on the edible part of lettuce in presence of exDNA quantities comparable to those possibly encountered in the agri-food system scenario. Moreover, the presented results indicate that after the acquisition of the plasmid pZR80(gfp), carrying an antibiotic resistance gene, transformant *A. baylyi* BD413 can enter the leaf tissues, and show that this ability is enhanced in the presence of heptamethyltrisiloxane, a surfactant adjuvant widely used in agriculture. The impact of agrochemicals on antibiotic resistance spread in agri-food systems is still overlooked and could be one of the aspects to consider for future research. All in all, we claim the importance to obtain more experimental data on HGT mechanisms directly in planta, since produces, together with animal-derived matrices, represent the possible entry point of ARGs and ARB into food production.

## Figures and Tables

**Table 1:** The ratio between survived and administered cells of the strains *A. baylyi* BD413, *E. coli* DH5α, and *K. cowanii* VR04.

Strain	1 h	24 h
*A. baylyi* BD413	9.39 × 10^−4^ ± 4.24 × 10^−4^	1.54 × 10^−4^ ± 5.11 × 10^−5^
*A. baylyi* BD413-endo	-	1.25 × 10^−6^ ± 3.29 × 10^−7^
*E. coli* DH5α	5.24 × 10^−3^ ± 3.87 × 10^−3^	4.34 × 10^−5^ ± 6.81 × 10^−5^
*K. cowanii* VR04	2.39 × 10^−2^ ± 1.19 × 10^−3^	3.01 × 10^−3^ ± 2.14 × 10^−3^

## Data Availability

The data presented in this study are available in the article and its supplementary material.

## Supplementary Materials

The following supporting information can be downloaded at: https://www.mdpi.com/article/10.3390/antibiotics11091231/s1. Figure S1: Confirmation of strain identity after the reisolation from lettuce leaves; Figure S2: Survival in a viable and culturable state shown by *A. baylyi* BD413, *E. coli* DH5α and *K. cowanii* VR04 on lettuce phylloplane; Figure S3: Confirmation of identity and plasmid acquisition by the putative *A. baylyi* BD413 transformants isolated from the leaf surface; Figure S4: Confirmation of identity and plasmid acquisition by the putative *A. baylyi* BD413 transformants isolated in the leaf endosphere; Figure S5: Cell membrane permeability of *A. baylyi* BD413 under the influence of heptamethyltrisiloxane (HPTSO); Figure S6: Plasmid DNA entry in the lettuce leaf tissues; Table S1: Assessment of the viable and culturable bacterial cells actually released by spray administration on lettuce leaves; Table S2: Comparison between the cell survival in a culturable state the shown by strains *A. baylyi* BD413, *E. coli* DH5α and *K. cowanii* VR04. Table S3: *A. baylyi* BD413 transformation frequency values on nitrocellulose membrane filters.
