# Supplementary material for: Acinetobacter baylyi Strain BD413 Can Acquire an Antibiotic Resistance Gene by Natural Transformation on Lettuce Phylloplane and Enter the Endosphere
Source: Antibiotics (Basel). 2022 Sep 10;11(9):1231. doi: 10.3390/antibiotics11091231 (PMC9495178; doi:10.3390/antibiotics11091231)
Supplement: Supplementary file 1 [file antibiotics-11-01231-s001.zip › antibiotics-1865968-supplementary.pdf]

Article

Acinetobacter baylyi Strain BD413 Can Acquire an Antibiotic Resistance Gene by Natural Transformation on Lettuce Phylloplane and Enter the Endosphere

Valentina Riva, Giovanni Patania, Francesco Riva, Lorenzo Vergani, Elena Crotti and Francesca Mapelli *

Department of Food, Environmental and Nutritional Sciences (DeFENS), University of Milan, 20133 Milan, Italy

* Correspondence: francesca.mapelli@unimi.it; Tel.: +39-02-50319115; Fax: +39-02-50319238

Table S1. Assessment of the viable and culturable bacterial cells actually released by spray administration on lettuce leaves. Concentration data are expressed as CFUs/ml, in triplicates, for the bacterial strains *A. baylyi BD413*, *E. coli* DH5α and *K. cowanii* VR04. Average values and standard deviations are reported for each strain below the replicate values. All strains were initially prepared as a cell suspension at the concentration of 10^8 cell/ml in a spray bottle.

**Bacterial strain**	**CFU/ml**
A. baylyi BD413-1	2.31×10^7
A. baylyi BD413-2	3.03×10^7
A. baylyi BD413-3	2.69×10^7
A. baylyi BD413	$2.68 \times 10^7 \pm 3.6 \times 10^6$
E. coli DH5α-1	3.44×10^7
E. coli DH5α-2	2.05×10^7
E. coli DH5α-3	4.35×10^7
E. coli DH5α	$3.28 \times 10^7 \pm 1.16 \times 10^7$
K. cowanii VR04-1	2.5×10^8
K. cowanii VR04-2	1.63×10^8
K. cowanii VR04-3	1.4×10^8
K. cowanii VR04	$1.84 \times 10^8 \pm 5.8 \times 10^7$

Table S2. Comparison between the cell survival in a culturable state shown by the strains *A. baylyi* BD413, *E. coli* DH5α and *K. cowanii* VR04. ANOVA (post-hoc Dunnett's test) has been conducted on the ratio between survived culturable cells and administered cells, calculated 1 hour and 24 hours after spray administration on lettuce leaves.

**1h**	**p-value**
BD413 vs DH5α	0.1126
BD413 vs VR04	<0.0001
**24h**	**p-value**
BD413 vs DH5α	0.9913
BD413 vs VR04	0.0521

Table S3. ***A. baylyi*** **BD413 transformation frequency values on nitrocellulose membrane filters.** The assay was conducted *in vitro* using the pZR80(gfp) plasmid at quantities comprised between 1 and 50 ng.

**Plasmid quantity (ng)**	**Transformation frequency**
1	$5.00 \times 10^{-5} \pm 4.29 \times 10^{-6}$
2	$6.71 \times 10^{-5} \pm 2.63 \times 10^{-6}$
5	$1.10 \times 10^{-4} \pm 1.83 \times 10^{-5}$
10	$1.28 \times 10^{-3} \pm 1.77 \times 10^{-4}$
20	$1.65 \times 10^{-3} \pm 1.22 \times 10^{-4}$
50	$1.74 \times 10^{-3} \pm 2.10 \times 10^{-5}$

Figure S1. Confirmation of strain identity after the reisolation from lettuce leaves. Agarose gel electrophoresis of ITS-PCR products of bacterial colonies isolated from lettuce leaf bacterized with (a-c) *A. baylyi* BD413, (d-e) *E. coli* DH5α RIF-R strain, (f-g) *K. cowanii* VR04 RIF-R strain. Panels refer to the colonies reisolated from the leaf surface 1h (a-d-f) or 24h (b-e-g) after bacterization, while (c) refers to *A. baylyi* BD413 colonies isolated from the leaf endosphere 24h after strain administration. PC = positive control (*i.e.*, ITS pattern of *A. baylyi* BD413, *E. coli* DH5α and *K. cowanii* VR04 strains); NC = negative control; M = marker.

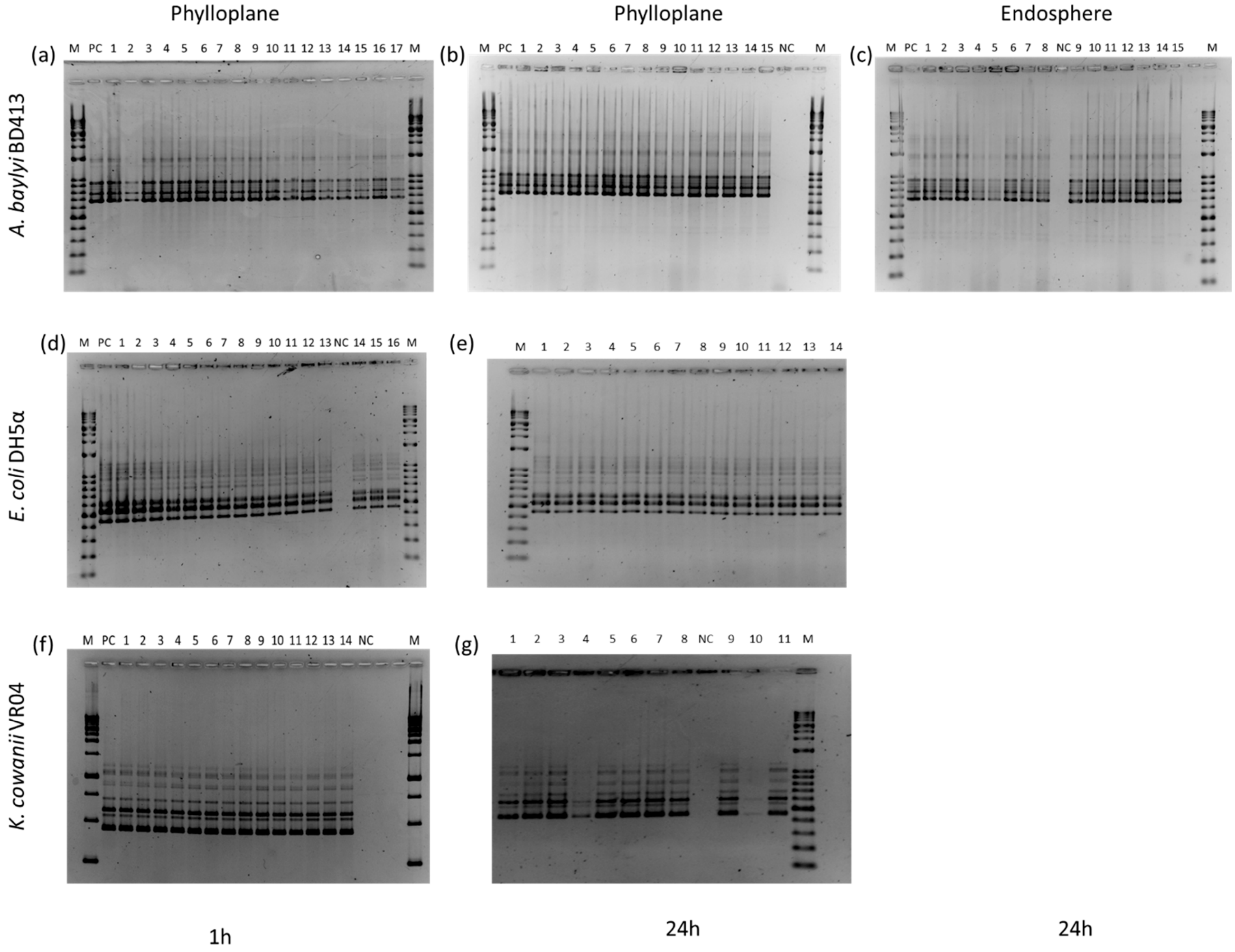

Figure S2. Survival in a viable and culturable state shown by *A. baylyi* BD413, *E. coli* DH5α and *K. cowanii* VR04 on lettuce phylloplane. For each strain, the ratio between the survived and the administered cells was assessed 1 hour (a) and 24 hours (b) after administration. The black line indicates the average value of three biological replicates. Different letters (a,b) indicate significant differences according to ANOVA (post-hoc Dunnett's test), where *A. baylyi* BD413 was considered as control thesis (p value < 0.0001).

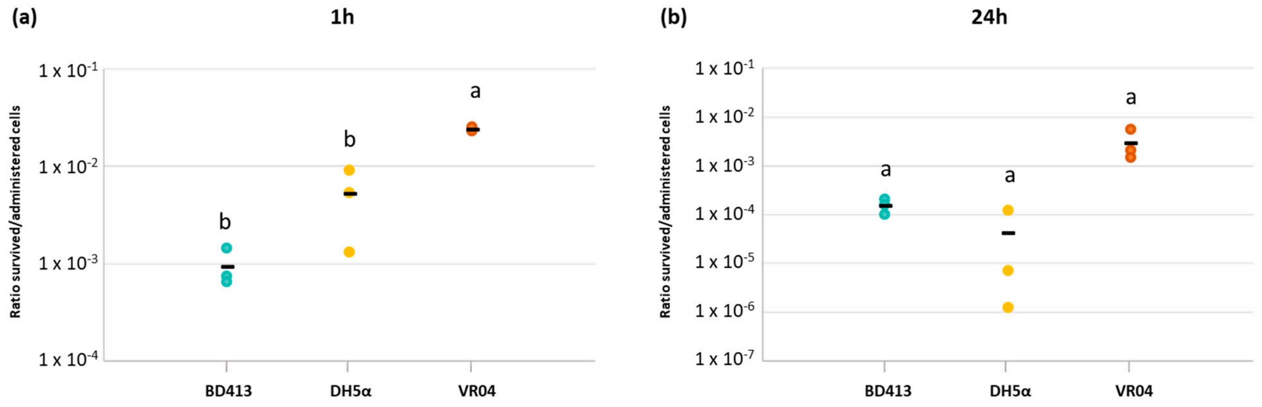

Figure S3. Confirmation of identity and plasmid acquisition by the putative *A. baylyi* BD413 transformants isolated from the leaf surface. Agarose gel electrophoresis of ITS-PCR (on the left side) and gfp-PCR (on the right side) products of transformant colonies reisolated from (a-b) leaf discs and (c-d) *in planta* at the end of natural transformation assays performed using 10 ng of exDNA. Panels (e-f) refer to the colonies reisolated from leaf discs using 1 ng of exDNA. Panel (g) shows representative images taken by phase-contrast and epifluorescence microscopy assessing the gfp gene expression by randomly selected transformant colonies (Scale bar = 6.8 µm). PC = positive control (*i.e.*, ITS pattern of *A. baylyi* BD413 and plasmid pZR80(gfp)); NC = negative control; M = marker.

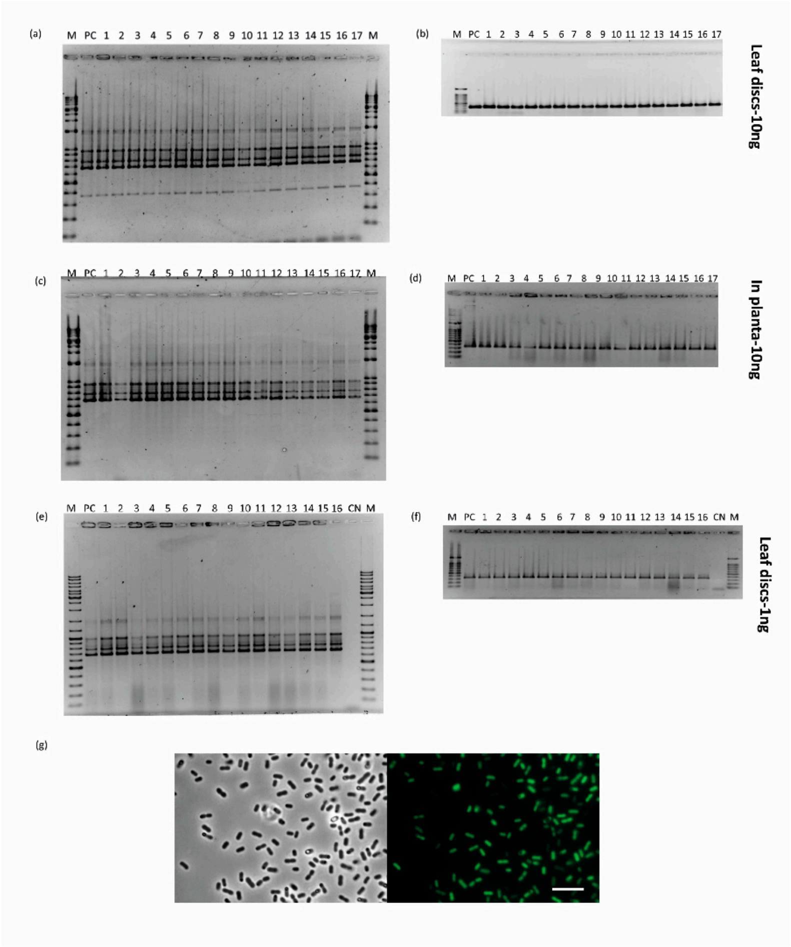

Figure S4. Confirmation of identity and plasmid acquisition by the putative *A. baylyi* BD413 transformants isolated in the leaf endosphere. Representative agarose gel electrophoresis of (a-b) ITS-PCR and (c-d) gfp-PCR products of transformant colonies reisolated from leaf endosphere in HPTSO presence (64 isolates, a-c) and absence (17 isolates, b-d). PC = positive control [*i.e.*, ITS pattern of *A. baylyi* BD413 and plasmid pZR80(gfp)]; NC = negative control; M = marker.

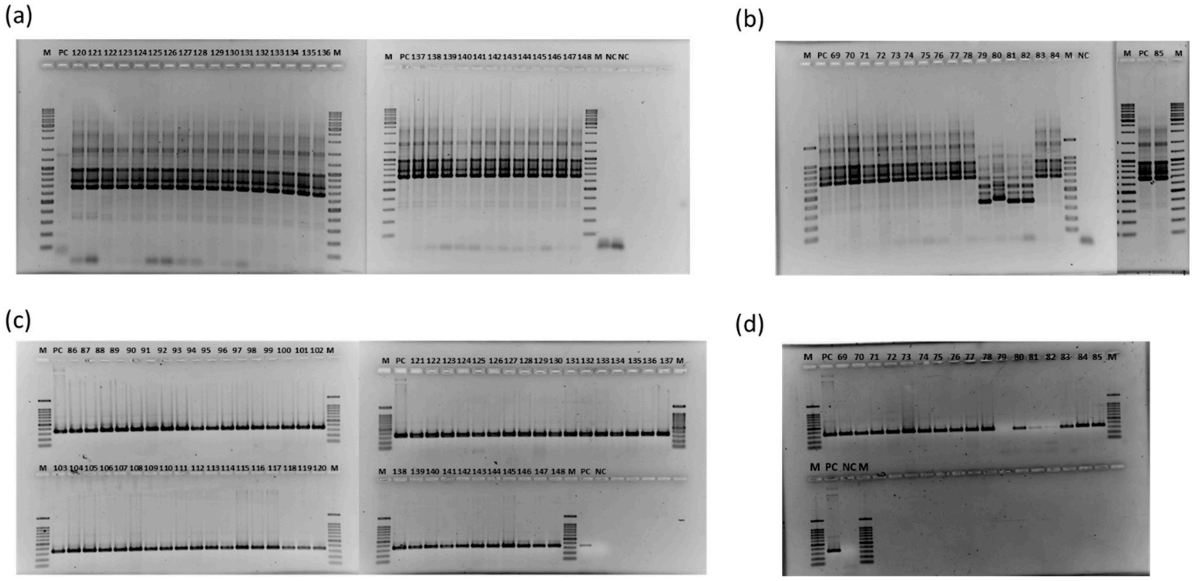

Figure S5. Cell membrane permeability of *A. baylyi* BD413 under the influence of heptamethyltrisiloxane (HPTSO). Inner membrane permeability assay (a). Total membrane permeability assay (b). For both tests, each data is the average of three biological replicates. According to Student's *t*-test, no statistically significant differences (p value > 0.05) between control and HPTSO treatments were detected at each considered time point.

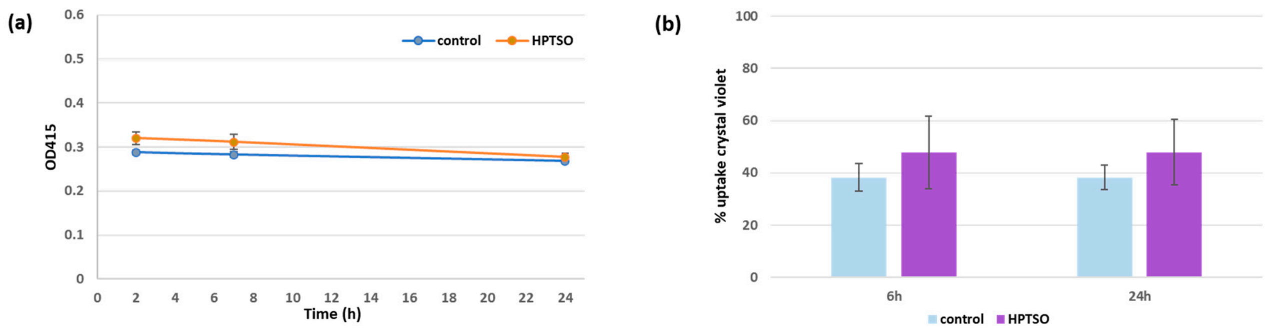

Figure S6. Plasmid DNA entry in the lettuce leaf tissues. Agarose gel electrophoresis of PCR amplified fragments of the genes *gfp* and *aphA* present on the pZR80(gfp) plasmid. The black arrow indicates the expected length of the fragment. 1-4: non-treated lettuce leaves; 5-8: +pZR80(gfp); 9-12: +pZR80(gfp) and +HPTSO; PC = positive control [*i.e.*, plasmid pZR80(gfp)]; NC = negative control; M = marker (a). qPCR evaluation of *gfp* copy numbers per gram of leaf tissue in non-treated control leaves (grey bar), leaves receiving the treatment with pZR80(gfp) (green bar) and those receiving the treatment with pZR80(gfp) and the HPTSO surfactant (orange bar). Stars indicate the significant differences (according to Dunnet's test) between the negative control and each treatment, confirming the *gfp* amplification in control samples as a background signal (b).

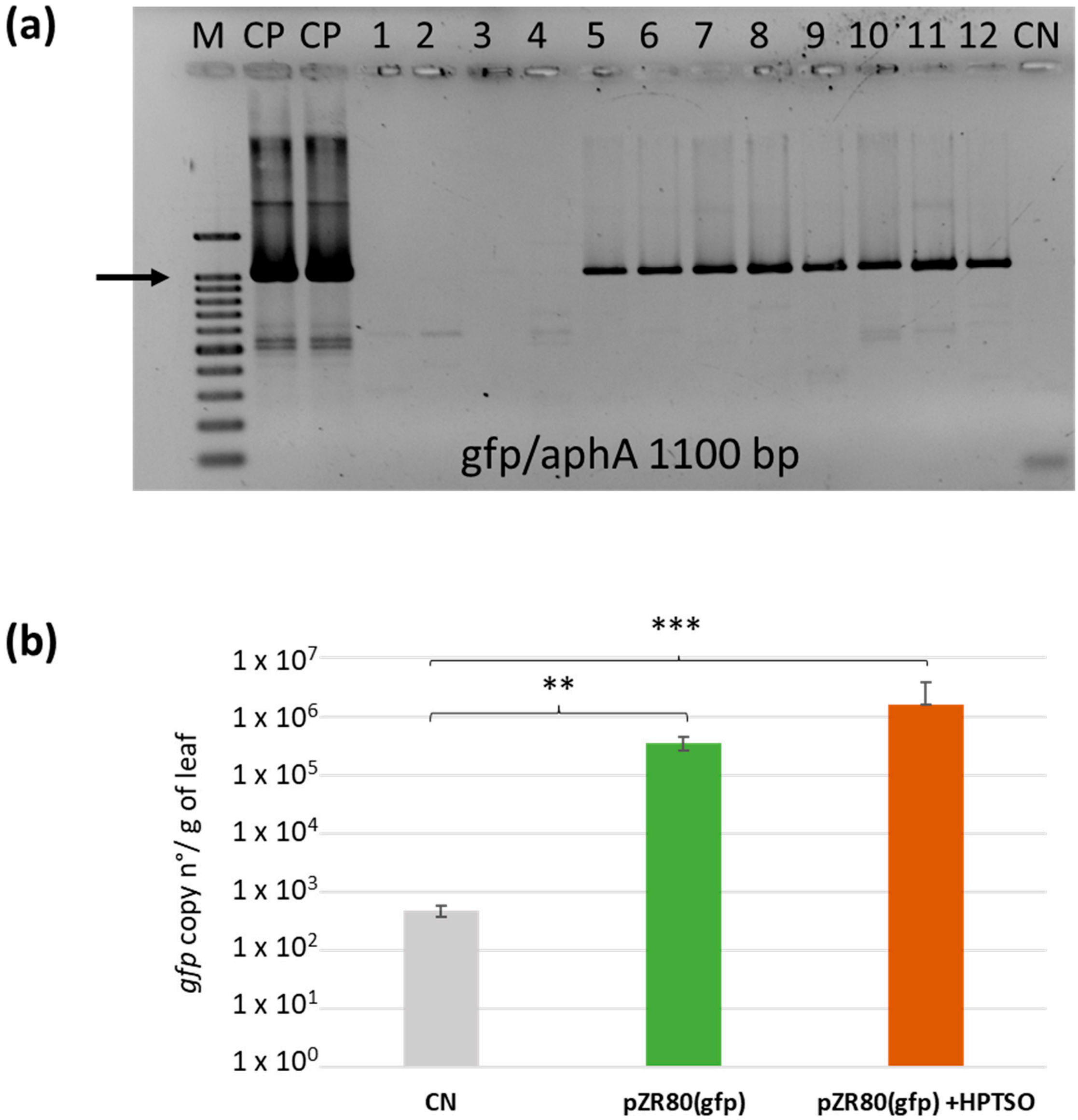
(a)
M CP CP 1 2 3 4 5 6 7 8 9 10 11 12 CN
gfp/aphA 1100 bp
(b)
***
**
1 x 10^7
1 x 10^6
1 x 10^5
1 x 10^4
1 x 10^3
1 x 10^2
1 x 10^1
1 x 10^0
gfp copy n°/ g of leaf
CN
pZR80(gfp)
pZR80(gfp) +HPTSO
